# The impact of urban regeneration programmes on health and health-related behaviour: Evaluation of the Dutch District Approach 6.5 years from the start

**DOI:** 10.1371/journal.pone.0177262

**Published:** 2017-05-09

**Authors:** Annemarie Ruijsbroek, Albert Wong, Anton E. Kunst, Carolien van den Brink, Hans A. M. van Oers, Mariël Droomers, Karien Stronks

**Affiliations:** 1 National Institute for Public Health and the Environment (RIVM), Bilthoven, The Netherlands; 2 Academic Medical Center (AMC), University of Amsterdam, Amsterdam, The Netherlands; 3 Tranzo, Faculty of Social Sciences, University of Tilburg, Tilburg, The Netherlands; National Institute of Child Health and Human Development, UNITED STATES

## Abstract

**Background:**

Large-scale regeneration programmes to improve the personal conditions and living circumstances in deprived areas may affect health and the lifestyle of the residents. Previous evaluations concluded that a large-scale urban regeneration programme in the Netherlands had some positive effects within 3.5 years. The aim of the current study was to evaluate the effects at the longer run.

**Methods:**

With a quasi-experimental research design we assessed changes in the prevalence of general health, mental health, physical activity, overweight, obesity, and smoking between the pre-intervention (2003–04 –mid 2008) and intervention period (mid 2008–2013–14) in 40 deprived target districts and comparably deprived control districts. We used the Difference-in-Difference (DiD) to assess programme impact. Additionally, we stratified analyses by sex and by the intensity of the regeneration programme.

**Results:**

Changes in health and health related behaviours from pre-intervention to the intervention period were about equally large in the target districts as in control districts. DiD impact estimates were inconsistent and not statistically significant. Sex differences in DiD estimates were not consistent or significant. Furthermore, DiD impact estimates were not consistently larger in target districts with more intensive intervention programmes.

**Conclusion:**

We found no evidence that this Dutch urban regeneration programme had an impact in the longer run on self-reported health and related behaviour at the area level.

## Introduction

Residents of deprived areas generally have worse health than those living in non-deprived areas. These health differences cannot be explained completely by individual characteristics, such as individual socioeconomic status [1]. The poorer living conditions in these deprived areas, for example a low quality housing stock, social disorder and deteriorated physical environment, may impact the mental and physical health of residents as well [2], thereby contributing to geographical health inequalities. These living conditions, together with the social position of people, make up the so-called social determinants of health [3]. Addressing these social determinants of health is important for tackling socioeconomic health inequalities [2].

Urban regeneration programmes aim to ameliorate the physical and social environment of deprived areas and may in addition address socioeconomic problems that are common in these areas, such as unemployment and high levels of school dropout. As these urban regeneration programmes target the social determinants of health, they may contribute to health improvements of residents and reduce geographical health inequalities [4,5]. The evaluation of the health impact of urban regeneration programmes can therefore provide information about the usefulness of area-level policies as a strategy to tackle poor health and health inequalities.

The Dutch District Approach is an urban regeneration programme that may impact the health of residents because it addressed the social determinants of health. The Dutch government launched this programme in 2007 with the aim to improve the living conditions of the 40 most deprived districts of the Netherlands within the next ten years [6]. Interventions were aimed at improving employment, educational level, housing conditions and the physical neighbourhood environment, safety, and social cohesion. Each district developed a set of locally tailored interventions. Examples of interventions include the renovation of the housing stock, programmes to prevent school dropout, debt restructuring, the creation of playgrounds and green space, programmes to reduce neighbourhood nuisance, and activities to increase the social cohesion, such as neighbourhood festivals. The mix, content and intensity of interventions differed per district [7]. Although the approach was intended to last 10 years, the national funding stopped in 2012 as a result of changes in political priorities [8].

A first evaluation of the health impact of the Dutch District Approach showed that for some health indicators trends in the ‘target districts’ were more favourable than in control districts [9–11], but overall there was no consistent evidence for a positive health impact of the urban regeneration programme [12]. The 3.5 years follow-up time of this initial evaluation might have been too short to establish conclusive evidence, because of the gradual processes that are involved. For example, effects on overweight can only be assessed after several years, as it may take some years before changes in physical activity or dietary patterns result into a reduced prevalence of overweight within populations. Indeed, evaluations in other countries often failed to show average health improvements on the short term [13–16], whereas studies evaluating the impact of urban regeneration after five or six years more often reported positive health results [17–20].

The initial evaluation of the Dutch District Approach showed the importance of considering specific population groups and selected districts [10,11]. For instance, a tendency towards a more positive trend in mental health in the target districts was found exclusively among female residents and in districts that implemented an intensive regeneration programme [10]. Studies from other countries underwrite the need for subgroup analyses [16–18,21].

In the current study, we examine the health impact of the Dutch District Approach up to 6.5 years after the introduction of the programme. We compare the health and health-related behaviour in the 40 target districts in the years following the start of the regeneration programme with the health and health-related behaviour levels in the years prior to the implementation. These changes are compared with changes in similarly deprived control districts, using a quasi-experimental approach. Furthermore, we examine whether the health impact differs by sex and by the intensity of the urban regeneration programme. We investigate the effect on general health, mental health, physical activity, smoking, overweight and obesity.

## Methods

### Dutch District Approach

In 2007, the Dutch Government launched the Dutch District Approach with the aim to improve the liveability in the 40 most deprived districts in the Netherlands, located in 18 large Dutch cities. These 40 districts were selected using registration data on physical and socio-economic deprivation, and survey information from residents concerning physical and social neighbourhood problems [22]. Besides funding, the national government provided support and expert advice. Local authorities were given autonomy to implement tailor-made interventions that fitted the specific local problems. The only requisite was that these interventions should fit in with the overall Dutch District Approach themes determined by the national government: employment, educational level, housing and the physical neighbourhood environment, safety and social cohesion. The local approaches varied in terms of the number of residents reached by the interventions, and the number and type of interventions implemented [7]. The implementation of interventions started in the course of 2008 in most instances. By 2012, around 5 billion euros had been spent to ameliorate problems addressing the five overall themes [23]. Since the termination of the national funding in 2012, many cities continued to intervene in the target districts, but at a much lower level of investment. In addition, on several occasions successful interventions from the district approach were implemented into other deprived districts in the city as well.

### Intensity of the urban regeneration programme

The previous evaluation of the Dutch District Approach included an inventory of the intensity of the regeneration programme in each district [7]. All activities within 17 distinguished types of action (grounded on the five overall themes) were listed for each district and scored, based on the number of participants reached or environmental change achieved (see S1 Table for an overview of the activities that were implemented, their level of impact, and the criteria for the scale of the activities). Next, each type of action was classified as ‘less intensive’, ‘moderately intensive’ or ‘more intensive’ activities [10]. For four target districts, detailed information on the activities was lacking and classification by intensity could not be made. For the remaining 36 districts, the average intensity score was calculated. We used this score to divide the districts into one group of 19 high intensity target districts with a more intense regeneration programme and another group of 17 target districts with a less intensive regeneration programme. See [10] for more detail on the classification.

### Study population

We used nationwide repeated cross-sectional health data from the Dutch Health Interview Survey (HIS), collected between January 2003 and December 2014 by Statistics Netherlands. Respondents are of all ages, living in private households in the Netherlands. Each month, Statistics Netherlands draws a person-based sample from the Dutch population register (approximately 15,000 persons per year). In the years 2003 to 2009, respondents were interviewed at home for the basic survey, which included questions about their general health, height, weight and smoking behaviour. A second, additional questionnaire was left behind after the interview for respondents older than 12 years to ask them about more sensitive topics, such as mental health, but also about their physical activity behaviour. Since 2010, the HIS employs a stepwise approach. First, the selected persons are asked to participate through internet. Second, non-respondents are approached and interviewed by telephone. Third, those who cannot be reached by internet or telephone are approached for a personal interview. The second, additional questionnaire could be filled out by internet or using a written questionnaire. In 2014, the basic survey and additional survey were combined into one survey that can be filled out either using the internet or through a personal interview. Between 2003 and 2014, the annual response rate for the main (in 2014 total) survey was 60–65%. Among those who responded, the response rate for the additional survey was around 80% (years 2003–2009) and around 55% (years 2010–2013). We selected data from adults who were aged 18 years and older and lived in the 40 target district or the control areas at January 1^st^ 2008. We selected adults, because we expected that the impact of the intervention programme might differ between adults and adolescents or children. Unfortunately, we had not enough respondents under the age of 18 years to examine this group separately.

### Matching on districts and individuals

We used a quasi-experimental design to investigate the effect of the Dutch District Approach on individual-level outcomes. We selected control districts similar to the target districts in terms of neighbourhood and individual characteristics. These characteristics were measured at January 1^st^ 2008, i.e. just prior to the start of the implementation, to ensure that the programme could not have influenced the characteristics, and ultimately the outcome. In matching target and control districts, we made the simplifying assumption that individuals did not migrate after 2008. The following is an outline of the matching steps; for details see S1 File.

Two steps were taken to ensure that the *joint* distribution of neighbourhood and individual characteristics were approximately the same in the target and control districts. (i.e. the probability of having a specific combination of neighbourhood characteristics *and* individual characteristics is roughly the same in each group.) First, we identified areas that were similar to the target districts with regard to their physical and social neighbourhood characteristics and safety (13 indicators) by means of propensity score matching (PSM). We used *subclassification on the propensity score* to reduce imbalance in neighbourhood characteristics between the target and control districts. For each target and potential control district, we estimated the propensity score as a function of neighbourhood-level characteristics, using logistic regression. We determined the *common support* of the areas, i.e. the range of propensity scores that was found in *both* the target and potential control districts, and discarded control districts that fell outside this range. Next, we divided the target districts into four subclasses. The control districts were divided over these four subclasses based on their propensity score. The target and control districts within one subclass had approximately the same propensity score and distribution of the neighbourhoods’ characteristics (see Table D in S1 File for the number of target and control districts per subclass). In total, 83 postal codes areas that constituted the 40 target districts and 182 control postal code areas remained in the study. The coverage of the target districts in our sample was complete in terms of postal codes.

Second, we selected *all* individuals from the Dutch Health Survey that lived in the target districts or the selected control districts at January 1^st^ 2008, and selected respondents within the control areas that were most similar to the residents in the target districts using *stratification* on individual characteristics, i.e. sex, age, educational level, household income and ethnicity. Per outcome measure, we used the most defining individual characteristics, because full stratification on all individual covariates or PSM on individual characteristics were not possible due to the small sample size (for the selected individual characteristics per outcome measure see Table E in S1 File). Each stratum contained individuals from target and control districts, who participated in the health survey in the pre-intervention period or intervention period.

For the estimation of the effect of high intensity and low intensity urban regeneration programmes, respectively, versus no programme, we could not use the previously described approach to adjust for neighbourhood level characteristics based on subclassification, because of the smaller sample size. Instead, we used 1:1 nearest neighbour matching on the propensity score to find a suitable control district for each target district. Stratification on individual characteristics was similar to the description above.

After the selection of respondents from the target and control districts, health data of 13,651 respondents were included in the analyses.

### Measures

We included the same health outcomes as used in the previous evaluation of the programme (i.e. general health, mental health, smoking, leisure-time walking, leisure-time cycling, and sport participation) and added overweight and obesity.

*General health* was measured using the following question: In general how is your health? Answers were dichotomised into good general health (good or very good) versus less than good general health (fine, bad or very bad).

*Mental health* was measured in the additional questionnaire using the Mental Health Inventory (MHI5), a measure with demonstrable reliability and validity [24]. MHI-5 assesses a person’s feelings of nervousness and depression during the past month, using questions with answers ranging from ‘all the time’ to ‘never’ on a six-point scale. Total sum scores were transformed into a scale from 0–100, with higher scores reflecting better mental health. Based on a Dutch validation study, we dichotomised this score into fair or good (>60) and less than fair or good (≤60) [25].

*Physical activity (PA)* was measured in the additional questionnaire as well, using the Dutch Short QUestionnaire to ASsess Health-enhancing physical activity (SQUASH). This instrument has shown to be fairly reliable and valid for measuring PA [26,27]. Three PA indicators were constructed, in accordance with the initial evaluation [8]. Total minutes per week spent on leisure time walking, leisure-time cycling, and sports were calculated by multiplying questions about duration (hours and minutes per week) and questions about frequency (days per week) and dichotomized into ‘inactive’ (0 minutes per week) versus ‘active’ (any minutes per week). Water related sports (e.g. skiing, surfing, and diving) were excluded from the analyses because these activities are strongly bound to spaces that were usually outside of residential areas. Agility sports (e.g. bowling, darts, golf) and mental sports (e.g. chess) were excluded from the analyses because their intensity was too low to be considered as intense PA.

*Smoking* was asked with a single question (Do you sometimes smoke?) and divided into smokers (yes) versus non-smokers /ex-smokers (no).

*Overweight* and *Obesity* were calculated based on the BMI (kg/m^2^) of the respondents, based on self-reported height (metres) and weight (kilograms). Respondents with a BMI of 25 or higher were labelled as overweight. Respondents with a BMI of 30 of higher were labelled as obese. The health outcome overweight also includes obesity.

For general health, smoking, overweight and obesity the pre-intervention period included the years 2003 to mid-2008 and the intervention period mid-2008 to 2014. For mental health and PA, we excluded data from 2014, because of methodological changes in the additional survey in 2014. To maintain balance in the pre-intervention and intervention period, we excluded data from 2003 as well, resulting in a pre-intervention period of 2004 to mid-2008 and intervention period of mid-2008 to 2013.

### Analyses

First, we obtained stratum-specific treatment effects by estimating the difference in outcome over time separately for the target and control districts per stratum and calculating the difference between these differences (Difference-in-Differences or DiD). The difference in outcome for the control group served as a way to control for unmeasured confounding (e.g. general trends in lifestyle or health) under the assumption that this affected the target and control districts similarly. We estimated stratum-specific treatment effects through a linear regression model, in which the outcome was estimated as a function of a period indicator (intervention period compared to its reference pre-intervention period), treatment indicator (treatment group, i.e. the target districts, compared to the reference group control) and the interaction between the period and treatment indicator. Next, the overall treatment effect was calculated by pooling the stratum-specific effects over all strata. The overall treatment effects were estimated in similar fashion for the high and low intensity urban regeneration programmes, with the main difference that the strata for the high/low intensity programme analyses were based on individual characteristics only. Sex-specific effects were estimated by pooling over strata concerning men and women, respectively.

## Results

Residents of the target areas were similar to the residents of the control districts in terms of age, sex and household composition, but were more likely to be lower educated, of non-western origin and with a lower income (Table 1).

**Table 1:** Characteristics of the study population ^a^.

	40 target districts		Control districts	
	Pre-intervention	Intervention	Pre-intervention	intervention
**4-digit postal codes**	**83**	**83**	**182**	**182**
**N respondents**	**2,319**	**1,864**	**5,129**	**4,339**
**Sex (% female)**	52.7	52.4	53.5	53.8
**Age (%)**				
18–35	38.9	34.9	36.0	31.4
35–55 years	31.3	35.7	29.8	35.1
55 years and older	29.8	29.4	34.2	33.6
**Household composition (%)**				
Single with/without child(ren)	39.2	40.5	36.7	37.8
Couples with/without child(ren), with others	60.8	59.5	63.3	62.2
**Ethnicity (%)**				
Ethnic Dutch, non-Dutch—Western	64.7	69.4	82.2	84.2
Non-Dutch, non-Western	35.3	30.6	17.8	15.8
**Education (%)**				
Primary, secondary	83.9	71.6	77.5	66.1
Tertiary	16.1	28.4	22.5	33.9
**Income (%)**				
First tertile (> €16,493)	40.4	35.3	32.8	30.1
Second tertile (€16,493 – €23,993)	34.1	34.8	33.3	32.5
Third tertile (> €23,993)	25.5	29.9	33.9	37.4

^a^ Characteristics represent average values over the period 2003 to 2014

During the whole study period, residents in the target districts more often reported poor health and unhealthy behaviour compared to residents in the control districts except for the prevalence of smoking in both periods and leisure time walking in the pre-intervention period, which were almost similar in the target and control districts (Table 2). Changes in health and health-related behaviour from pre-intervention to the intervention period were about equally large in the target and control districts and the DiD impact estimates were inconsistent and non-significant.

**Table 2:** Comparison of health and health-related behaviour between 2003- mid 2008 and mid 2008–2014 (general health, smoking, overweight and obesity) and between 2004–2008 and 2009–2013 (mental health, leisure-time walking, leisure-time cycling and weekly sports) in 40 target districts and the control group.

		Target districts			Control districts				
		Pre-intervention	Intervention	Intervention versus pre-intervention period^a^	Pre-intervention	Intervention	Intervention versus pre-intervention period^a^	DiD (C.I.)^c^	p-value
	*n*^b^	*%*	*%*	*%*	*%*	*%*	*%*		
Good general health	9,900	68.0	66.2	-1.8	74.3	72.8	-1.5	-0.2 (-5.5;5.5)	0.94
Fair or good mental health	5,431	83.0	82.8	-0.2	86.8	85.9	-0.9	0.7 (-5.3;6.7)	0.81
Leisure-time walking	5,679	60.1	64.0	3.9	60.4	66.3	5.9	-2.0 (-9.9;5.8)	0.62
Leisure-time cycling	5,431	41.5	45.0	3.5	47.7	52.7	5.0	-1.5 (-10.2;7.2)	0.73
Sports participation	5,782	40.0	38.2	-1.8	45.7	41.7	-4.0	2.3 (-5.8;10.4)	0.58
Overweight	8,853	45.1	50.3	5.2	41.3	45.2	3.9	1.3 (-5.2;7.7)	0.70
Obesity	8,853	14.8	15.0	0.2	10.4	12.5	2.1	-1.9 (-6.2;2.4)	0.40
Smoking	9,889	33.1	31.5	-1.6	34.5	32.6	-1.9	0.2 (-5.5;5.9)	0.94

^a^ Reference category

^b^ The n is the sum of all four groups used in the analysis

^c^ Difference in Difference (Confidence Intervals)

Table 3 shows the changes in health and health-related behaviours between the pre-intervention and intervention period and the DiD impact estimates separately for men and women. There is no consistently different pattern in the DiD impact estimates between men and women. For female residents, a tendency to more favourable changes in mental health, overweight and obesity between the pre-intervention and intervention period in the target districts compared with the control areas was found, which was not found among men. On the other hand, men living in the target districts had a tendency for more favourable changes in smoking and sport participation compared with men in the control areas, which was not found among women. In all instances, the difference in changes in health and health-related behaviour between the target and control districts remained insignificant in both sexes.

**Table 3:** Comparison of health and health-related behaviour between 2003- mid 2008 and mid 2008–2014 (general health, smoking, overweight and obesity) and between 2004-mid 2008 and mid 2008–2013 (mental health, leisure-time walking and cycling, and weekly sports) in 40 target districts and the control districts, stratified by sex.

		Target districts			Control districts				
		Pre-intervention	Intervention	Intervention versus pre-intervention^a^	Pre-intervention	Intervention	Intervention versus pre-intervention^a^	DiD (C.I.)^c^	p-value
sex	*n*^b^	*%*	*%*	*%*	*%*	*%*	*%*		
**Good general health**									
male	4,641	70.0	67.7	-2.3	78.5	75.2	-3.3	1.0 (-6.3;8.4)	0.78
female	5,213	65.9	65.7	-0.2	71.1	71.1	0.0	-0.3 (-8.2;7.6)	0.95
**Fair or good mental health**									
male	2,526	88.4	84.1	-4.3	91.6	89.6	-2.0	-2.3 (-9.8;5.2)	0.55
female	2,905	78.3	81.7	3.4	82.6	82.6	0.0	3.4 (-5.7;12.4)	0.47
**Leisure-time walking**									
male	2,605	59.5	63.1	3.6	58.5	62.9	4.4	-0.8 (-12.2;10.7)	0.90
female	3,074	60.7	64.4	3.7	62.1	69.2	7.1	-3.3 (-14.1;7.4)	0.55
**Leisure-time cycling**									
male	2,337	43.0	46.6	3.6	48.9	55.5	6.6	-3.1 (16.6;10.5)	0.66
female	2,696	42.3	46.1	3.8	47.5	52.7	5.2	-1.3 (-13.5;10.8)	0.83
**Sport participation**									
male	2,494	39.7	42.7	3.0	44.3	43.6	-0.7	3.7 (-8.1;15.5)	0.54
female	3,020	41.1	34.6	-6.5	46.4	39.7	-6.7	0.1 (-10.7;11.0)	0.98
**Overweight**									
male	4,164	48.1	56.7	8.6	46.1	48.7	2.6	6.0 (-3.3;15.4)	0.21
female	4,675	43.2	43.3	-0.1	36.6	42.1	5.5	-5.4 (-14.4;3.7)	0.24
**Obesity**									
male	4,164	13.2	14.7	1.5	9.3	10.7	1.4	0.1 (-5.7;6.0)	0.97
female	4,675	16.9	15.0	-1.9	11.7	14.3	2.6	-4.4 (-10.8;2.0)	0.18
**Smoking**									
male	4,629	40.7	36.8	-3.9	38.9	38.4	-0.5	-3.5 (-12.0;5.0)	0.41
female	5,260	26.4	26.7	0.3	30.7	27.5	-3.2	3.5 (-4.3;11.3)	0.38

^a^ Reference category

^b^ The n is the sum of all four groups used in the analysis

^c^ Difference in Difference (Confidence Intervals)

Additionally, we examined the DiD impact estimates separately for high and low intensity target districts, but found no significant effects of the regeneration programme in either of these two groups (S2 Table).

Finally, in order to examine whether effects would occur when only the later intervention years were considered, we additionally divided the intervention period into an early (2008–2011) and late (2012-2013/2014) impact period, but found no significant impact of the regeneration programme either (S3 Table).

## Discussion

We examined the changes in health and health related behaviour during and following the implementation of a Dutch urban regeneration programme in 40 deprived districts. Our longer follow-up time (up to 6.5 years) compared to earlier evaluations allowed us to examine the longer-term health effects of this programme. The DiD impact estimates were inconsistent and not statistically significant, i.e. we did not observe significantly different changes in health or health-related behaviour in the 6.5 years after the start of the programme in the target districts compared to the control districts. There was no significant or consistently different pattern in the DiD impact estimates between men and women. Furthermore, DiD estimates were not consistently larger in target districts with a more intensive intervention programme.

### Limitations of the study

Several limitations of our study should be mentioned before we discuss the findings. Although we used propensity score matching (PSM) to select our control areas, the target and control districts still differed in terms of their deprivation levels. Because the Dutch District Approach aimed to address the most deprived areas in the Netherlands, it was difficult to find equally deprived areas for our control group. On average, it is fair to state that the control districts were less deprived and the problems in these districts were less severe than in the target districts. Dissimilarity of our control areas could have biased our findings to some extent.

Because of the relatively small number of respondents in our study, we were limited in the number of individual characteristics we could use for stratification at the individual level. This did not seem to play a major role, however. Sensitivity analyses showed that controlling for these individual characteristics had only marginal effect on our estimates, suggesting that stratification on all potentially relevant individual characteristics would have had an equally limited impact on the estimates.

During our study period, social and physical interventions have most likely been implemented in the control districts as well. These interventions could have contributed to an underestimation of the intervention effect as it affected our case-control design. However, the investments in the 40 target districts were considerably larger and more comprehensive than the activities implemented concurrently in other deprived districts [28]. We therefore consider it to be unlikely that the interventions that took place in the control districts have affected our evaluation to the extent that rendered us unable to detect any existing health effects of the Dutch District Approach.

Our study comprised the adult population; not children or adolescents. This is unfortunate, since a substantial part of the interventions included the school environment and school system. We could not evaluate the possibility that the regeneration programme impacted the health of the younger population positively.

Finally, in our study design we excluded respondents who moved into the target and control districts after 2008 to prevent that potential positive health effects are caused by the inward migration of healthier residents. As a result, mobility patterns inwards could not have affected our findings. Outward migration, on the other hand, could have. For the individuals in the period 2006–2008, 18.2% of all individuals in control districts and 19.5% of all individuals in target districts have moved out at some point during 2009–2014. Outward migration to dissimilar districts (that is, individuals from target districts moving to non-target districts and individuals from control districts moving to non-control districts) could have affected our results, as it might decrease the actual difference between the two groups in exposure to the intervention. In that case, the health impact of the Dutch District Approach would be underestimated. However, such underestimation is probably mitigated, because we applied a matching method across target and control districts, as well as over time, and we thus controlled as much as possible for any differences in population characteristics across districts and over time.

### Discussion of the findings

In the current study, with a maximum follow-up time of 6.5 years, we did not observe a health impact of the Dutch District Approach. Three reasons might possibly explain these findings.

Firstly, the programme duration was relatively short, whilst these types of intervention programmes may need considerable time and funding to become effective [19]. The Dutch District Approach intended to take place during ten years, until 2017, but the national programme stopped prematurely and the national funding lasted only for four years, until 2011 [28]. As a result, from 2012 onwards the *extra* efforts that took place in most target districts sobered down or stopped. The resulting four-year programme of the Dutch District Approach might have been too short to induce noticeable health effects. An evaluation of the New Deal for Communities (NDC) regeneration programme in the U.K. suggested that time frames of at least five or six years are needed for these programme to exert a noticeable impact [19].

Secondly, health was not one of the five goals defined at the start of the programme. We hypothesised that the health of the residents could improve over time, because this regeneration programme addressed various social determinants of health. However, maybe health goals should be made explicit in this kind of complex regeneration programmes to make sure that the interventions and activities selected to improve any of the themes of the District Approach will benefit (or at least not harm) health and related behaviour. A Health-in-All-Policies approach (HiAP), where health is an explicit aim within other policy fields, may have led to other activities and maybe other results.

Thirdly, the interventions that were implemented in the four years of the Dutch District Approach were perhaps not intensive enough to induce a noticeable health impact. A Dutch study that evaluated the programme’s primary outcomes reported that changes in liveability, social cohesion, safety, and social mobility in the 40 target districts were generally similar to comparably deprived control districts [28]. Without clear positive changes in the social determinants of health, one cannot expect large health impacts either. According to this Dutch evaluation of the primary outcomes [28], the large ambitions of the programme might have led to inefficiencies. For instance the considerable amount of energy it took to choose local priorities within the larger programme and the fragmentation of activities due to the broadly defined goals could have undermined the success of the programme.

We did not find health effects of the Dutch District Approach at the area level, but this does not mean that there were no health benefits among some individuals. Our analyses of the treatment group (the target districts) included all respondents living in the districts, instead of only those residents who may have benefitted from activities specifically targeted to them. The evaluation of the New Deal for Communities (NDC) in the UK found no substantial effects in community outcomes at the area level. However, residents who participated in NDC activities reported greater changes in community outcomes than those who did not get involved [29]. In addition, in Australia, health improvements associated with neighbourhood renewal were only observed among those residents who participated in the renewal programme [16].

### Conclusion

We found no evidence that the Dutch District Approach had an impact in the longer run on self-reported health and related behaviour at the area level.

## Supporting information

S1 File Propensity score matching and stratification to define the control group. (DOCX)

S1 Table Type of activities, level of impact, and criteria for a smaller, intermediate, or larger scale of the combined activities from the Dutch District Approach. (DOCX)

S2 Table Comparison of health and health-related behaviour in low intensity target districts, high intensity target districts and control districts. (DOCX)

S3 Table Comparison of health and health-related behaviour between the pre-intervention period and the early and late intervention period. (DOCX)
