# Supplementary material for: The impact of urban regeneration programmes on health and health-related behaviour: Evaluation of the Dutch District Approach 6.5 years from the start
Source: PLoS One. 2017 May 9;12(5):e0177262. doi: 10.1371/journal.pone.0177262 (PMC5423649; doi:10.1371/journal.pone.0177262)
Supplement: S1 File — (DOCX) [file pone.0177262.s001.docx]

**S1 Propensity Score Matching and stratification to define the control group**

In contrast to experiments, treatment in observational studies is not randomly assigned by the researcher, and systematic differences will likely occur between those who receive a treatment (here the target districts), and those who do not (the control districts). In the current study, systematic differences could exist on two levels; at the neighbourhood level, where living conditions may differ between districts, and at the individual level, where the demographic composition of individuals may vary between districts. To consider systematic differences at the neighbourhood and individual level, we applied matching methods [1].

*The first stage: using the propensity score as a balancing score*

We used propensity score matching (PSM) to adjust for confounding on the neighbourhood level. The propensity score is defined as the probability of treatment for a given unit; in our case we define it as the probability of treatment for a district given its neighbourhood characteristics, and we estimate it with logistic regression. The propensity score works as a *balancing score*: after applying a matching method on the propensity score, the joint distribution of covariates will be approximately the same within the treatment and control group [2], which then allows for treatment effect estimation. However, this assumes that the following assumptions have been met: (1) the Stable Unit Treatment Value Assumption (SUTVA) and (2) the Strong Ignorability assumption.

The first assumption states that the outcome of one unit cannot be affected by the treatment of other units. An example of a violation of SUTVA would be that if investments are made to increase the safety at the edge of a given district A, then these investments may have a side effect in the form of increasing safety in district B that borders on A. While we cannot rule out such spillover effects, if such presents are present, these effects are likely to be found at the edge(s) of district B, making the impact modest on the outcome in district B.

The second assumption states that conditional on the observed covariates, the treatment assignment is random – or in other words, does not depend on unobserved covariates. In practice, this assumption can rarely be proven, and its plausibility can only be argued through considering which potential confounders are missing and their effects on the treatment assignment. In our case, this assumption seems plausible as we include a wide variety of characteristics that describe the living conditions within a neighbourhood, and also may influence the treatment assignment (in real life, by the government) and the outcome (variables related to health and lifestyle on the individual level). These characteristics concern the *district’s housing conditions* (the total number of houses, the number of housing withdrawals, and the share of compact housing, old housing, social housing units, apartments, respectively) and the *experiences of the district’s inhabitants* (percentage satisfied with current residence, satisfied with current surroundings, having encountered graffiti on walls and buildings, having encountered vandalism, experiencing nuisance by neighbours, experiencing nuisance by residents, experiencing fear for being robbed or harassed). Descriptive statistics for the estimated propensity score are listed below:

**Table A: Descriptive statistics for the estimated propensity score by treated and control group**

|  | **Minimum** | **1^st^ quartile** | **Median** | **Mean** | **3^rd^ quartile** | **Maximum** |
| --- | --- | --- | --- | --- | --- | --- |
| Treated | 0.01975 | 0.38820 | 0.62270 | 0.60020 | 0.85280 | 0.99910 |
| Control | 0.0000000 | 0.0000018 | 0.0000100 | 0.0099710 | 0.0001305 | 0.9665000 |

We find that the mean propensity score (rounded) in the treated group is 0.600, and 0.010 in the control group, which implies there the treated and control groups are strongly unbalanced in terms of neighbourhood characteristics. The 3^rd^ quartile of the propensity score for the control group is 0.0001, which means that 75% of the control districts have a lower (estimated) propensity score than this value. This implies that the majority of control districts have very little chance of being selected as a target district, given their characteristics. In other words, the majority of the control districts are not a priori comparable with the target districts, underlines the need for matching methods.

*The first stage continued: reducing imbalance on the neighbourhood characteristics*

After estimating the propensity score, we determined the *common support* (i.e. the range of propensity score that was found both in the treated and control districts) and the discarded the control districts that fell below this range (<0.01975). The rationale behind this is that the control districts with a very low propensity score are generally much less deprived, and in that sense not comparable with the target districts. In total, 83 target districts and 182 control districts remained after this step.

Then we used subclassification on the propensity score to reduce the imbalance in the district characteristics between treated and control groups. This implies dividing districts into four subclasses based on the propensity score, such that treated and control districts that fall within a subclass had approximately the same propensity score and distribution of the neighbourhoods’ characteristics, and such that the number of treated districts was roughly the same in each subclass. The use of such subclasses allow us to sample individuals by subclass in the second stage of matching (see below), mimicking the aforementioned experimental design that was described in the methods section. The sample sizes of each subclass are reported below:

**Table B: Descriptives of the initial subclasses formed for subclassification on the propensity score**

|  | **Subclass 1** | **Subclass 2** | **Subclass 3** | **Subclass 4** | **Subclass 5** |
| --- | --- | --- | --- | --- | --- |
| Average propensity score | 0.089 | 0.411 | 0.633 | 0.810 | 0.959 |
| Treated | 17 | 16 | 17 | 16 | 17 |
| Control | 146 | 21 | 7 | 5 | 3 |
| Total | 163 | 37 | 24 | 21 | 20 |

After subclassification on the propensity score, the covariate imbalance has reduced strongly. Below the standardized differences in means between treatment and control groups (weighted over all subclasses) are reported before and after matching for each covariate.

**Table C: Balance results before and after subclassification on the propensity score**

|  | **Before subclassification** | | | **After subclassification** | | |
| --- | --- | --- | --- | --- | --- | --- |
|  | **Means treatment** | **Means control** | **Standardized difference** | **Means treatment** | **Means control** | **Standardized difference** |
| Distance in propensity score | 0.592 | 0.186 | 1.436 | 0.592 | 0.555 | 0.068 |
| % compact housing | 0.329 | 0.292 | 0.474 | 0.329 | 0.314 | 0.217 |
| % apartment | 0.699 | 0.581 | 0.584 | 0.699 | 0.662 | 0.236 |
| % old housing | 0.716 | 0.684 | 0.249 | 0.716 | 0.708 | 0.073 |
| % social housing | 0.599 | 0.494 | 0.745 | 0.599 | 0.589 | 0.131 |
| No. houses | 4477.578 | 4075.775 | 0.212 | 4477.578 | 3992.349 | 0.289 |
| No. withdrawals | 58.831 | 31.516 | 0.281 | 58.831 | 48.621 | 0.116 |
| % satisfied with residence | 73.949 | 81.229 | -0.723 | 73.949 | 72.088 | 0.284 |
| % satisfied with surroundings | 63.562 | 70.222 | -0.619 | 63.562 | 61.785 | 0.323 |
| % graffiti | 32.831 | 29.13 | 0.292 | 32.831 | 31.749 | 0.305 |
| % vandalism | 37.511 | 31.428 | 0.419 | 37.511 | 35.096 | 0.275 |
| % nuisance neighbours | 30.016 | 28.492 | 0.185 | 30.016 | 30.752 | 0.324 |
| % nuisance residents | 38.645 | 35.506 | 0.316 | 38.645 | 39.971 | 0.221 |
| % fear of being robbed or bothered | 34.509 | 26.673 | 0.682 | 34.509 | 30.525 | 0.23 |

Because subclass 5 contains very few control districts, making estimation of the treatment effect therein difficult, we pooled subclass 4 and 5:

**Table D: Descriptives of the revised subclasses for subclassification on the propensity score**

|  | **Subclass 1** | **Subclass 2** | **Subclass 3** | **Subclass 4** |
| --- | --- | --- | --- | --- |
| Average propensity score | 0.089 | 0.411 | 0.633 | 0.883 |
| Treated | 17 | 16 | 17 | 33 |
| Control | 146 | 21 | 7 | 8 |
| Total | 163 | 37 | 24 | 41 |

*The second stage: balance on individual characteristics*

Subclassification on the propensity score on individual level did not lead to satisfying balance on individual characteristics, so we used exact stratification on the individual characteristics. Since (a) we have 6 characteristics (age, sex, household income, education, the presence of a partner and ethnicity) with a theoretical number of $3^{2}\times2^{4}=144$ strata possible, (b) relatively small sample sizes, and (c) stratum-specific treatment effects can only be estimated if there are sufficient observations are present for that stratum, full stratification was not feasible. Instead, we opted to only stratify on (a maximum of) three covariates that are most relevant to the outcome. We used the random forests method (see [3]) to determine which the most important covariates per outcome were. Random Forests is a non-parametric classification and regression method that yields estimates that are robust to noise, and also yields variables importance rankings. A variable is considered most important if the prediction accuracy drops the strongest after permuting this variable. We select the top three variables for each outcome at most; less if fewer than three variables lead to a (relatively) considerable drop in prediction accuracy. The table below lists the variables used for stratification for each outcome:

**Table E: Individual characteristics used for stratification on individual level**

| **Outcome** | **Covariate 1** | **Covariate 2** | **Covariate 3** |
| --- | --- | --- | --- |
| General health | Age | Household income | - |
| Smoking | Age | Education | Sex |
| BMI | Age | Education | - |
| Mental Health | Household income | Age | Sex |
| Walking | Age | - | - |
| Biking | Ethnicity | Education | Age |
| Doing sports | Age | Education | Household income |

*Estimating treatment effects*

After forming strata, we fit a linear regression model on each stratum in which at least two observations are found for each treatment (urban renewal program) and time category (2003-2008 / 2009-2014):

$$Y_{s}=\beta_{s,0}+\beta_{s,1}\times Treatment+\beta_{s,2\times}Time+\delta_{s}Treatment\times Time,$$

where $s$ denotes a stratum, $Y_{s}$ is the outcome, $Treatment$ is an indicator function (0=control, 1= treated), and $Time$ also an indicator function (0=pre-intervention, 1=post-intervention). $\beta_{s,0}$, $\beta_{s,1}$ , $\beta_{s,2}$ and $\delta_{s}$ are the parameters to be estimated, with $\delta_{s}$ being the stratum-specific treatment effect.

Following [4], the point estimate for the *overall* treatment effect $\delta$ can be estimated by taking a weighted estimate

$$E[\delta]=\sum_{s=1}^{S} \frac{N_{s}}{N}\delta_{s}$$

With the weights being defined the share of the number individuals $N_{s}$in stratum $s$ in the total population size $N$. The variance of the estimate of $\delta$, $E[\delta]$ , is then:

$$Var(E[\delta])=Var\left( \sum_{s=1}^{S} \frac{N_{s}}{N}\delta_{s} \right)=Var\left( \frac{N_{1}}{N}\delta_{1} \right)+\ldots+Var\left( \frac{N_{S}}{N}\delta_{S} \right)=\left( \frac{N_{1}}{N} \right)^{2}Var\left( \delta_{1} \right)+\ldots+\left( \frac{N_{S}}{N} \right)^{2}Var\left( \delta_{S} \right)$$

$$=\sum_{s=1}^{S} \left( \frac{N_{s}}{N} \right)^{2}Var\left( \delta_{s} \right)$$

*The effect of high and low intensity programs*

We also estimated the effects of high intensity and low intensity urban renewal programs, respectively, versus no program. We once again use the propensity score to achieve balance between different groups here, but two alternative propensity scores definitions are used here; (1) the probability of *high* intensity programs versus no renewal program, and (2) the probability of *low* intensity programs versus no renewal program.

Because of the smaller sample size, the previously described approach to adjust for neighborhood level characteristics based on subclassification did not lead to a satisfactory improvement in balance. Instead, we used 1:1 nearest neighbor matching on the propensity score to find a suitable control districts to each treatment district, and using the entire resulting matched districts to sample individuals and stratify them on individual characteristics. This implies adjustment for neighborhood characteristics is done exclusively through the discarding of dissimilar control neighborhoods. Below the balance improvements for the high and low intensity renewal programs are given. In both cases the balance has improved greatly after matching, but some differences between the two groups still remain.

After matching on the neighborhood level, we proceed to estimate treatment effects in similar fashion to the estimation of the main intervention effect (see above).

**Table F: Balance of neighborhood characteristics: Low intensity versus control**

|  | **Before nearest neighbour matching** | | | **After nearest neighbour matching** | | |
| --- | --- | --- | --- | --- | --- | --- |
|  | **Means treatment** | **Means control** | **Difference** | **Means treatment** | **Means control** | **Difference** |
| Distance in propensity score | 0.41 | 0.004 | 0.406 | 0.41 | 0.305 | 0.105 |
| % compact housing | 0.31 | 0.155 | 0.155 | 0.31 | 0.302 | 0.008 |
| % apartment | 0.621 | 0.173 | 0.448 | 0.621 | 0.644 | -0.023 |
| % old housing | 0.71 | 0.5 | 0.21 | 0.71 | 0.706 | 0.004 |
| % social housing | 0.618 | 0.238 | 0.38 | 0.618 | 0.611 | 0.007 |
| No. houses | 4194.773 | 1984.414 | 2210.359 | 4194.773 | 4627 | -432.227 |
| No. withdrawals | 72.5 | 5.104 | 67.396 | 72.5 | 78.864 | -6.364 |
| % satisfied with residence | 74.296 | 92.99 | -18.694 | 74.296 | 77.915 | -3.619 |
| % satisfied with surroundings | 61.885 | 88.815 | -26.93 | 61.885 | 68.725 | -6.84 |
| % graffiti | 31.29 | 9.55 | 21.74 | 31.29 | 35.137 | -3.847 |
| % vandalism | 37.909 | 17.586 | 20.323 | 37.909 | 40.089 | -2.18 |
| % nuisance neighbors | 26.421 | 12.831 | 13.59 | 26.421 | 26.651 | -0.23 |
| % nuisance residents | 38.42 | 14.103 | 24.317 | 38.42 | 39.256 | -0.836 |
| % fear of being robbed or bothered | 34.204 | 7.965 | 26.239 | 34.204 | 32.091 | 2.113 |

**Table G: Balance of neighborhood characteristics: High intensity versus control**

|  | **Before nearest neighbour matching** | | | **After nearest neighbour matching** | | |
| --- | --- | --- | --- | --- | --- | --- |
|  | **Means treatment** | **Means control** | **Difference** | **Means treatment** | **Means control** | **Difference** |
| Distance in propensity score | 0.502 | 0.007 | 0,495 | 0.502 | 0.331 | 0,171 |
| % compact housing | 0.332 | 0.155 | 0,177 | 0.332 | 0.326 | 0,006 |
| % apartment | 0.709 | 0.173 | 0,536 | 0.709 | 0.685 | 0,024 |
| % old housing | 0.722 | 0.5 | 0,222 | 0.722 | 0.687 | 0,035 |
| % social housing | 0.614 | 0.238 | 0,376 | 0.614 | 0.597 | 0,017 |
| No. houses | 4522.688 | 1984.414 | 2538,274 | 4522.688 | 4467.021 | 55,667 |
| No. withdrawals | 68.167 | 5.104 | 63,063 | 68.167 | 62.667 | 5,5 |
| % satisfied with residence | 73.857 | 92.99 | -19,133 | 73.857 | 76.065 | -2,208 |
| % satisfied with surroundings | 64.945 | 88.815 | -23,87 | 64.945 | 67.879 | -2,934 |
| % graffiti | 32.47 | 9.55 | 22,92 | 32.47 | 33.726 | -1,256 |
| % vandalism | 36.303 | 17.586 | 18,717 | 36.303 | 35.335 | 0,968 |
| % nuisance neighbors | 29.922 | 12.831 | 17,091 | 29.922 | 29.262 | 0,66 |
| % nuisance residents | 36.766 | 14.103 | 22,663 | 36.766 | 33.689 | 3,077 |
| % fear of being robbed or bothered | 33.377 | 7.965 | 25,412 | 33.377 | 29.719 | 3,658 |

**References**

1. Stuart, E.A. Matching methods for causal inference: A review and a look forward. Stat Sci. 2010; 25(1): 1–21

2. Rosenbaum, PR. Observational Studies. New York: Springer-Verlag; 2002

3. Breiman, L. Random Forests. Machine Learning. 2001; 45(1): 5-32

4. Lunceford, JK, Davidian, M. Stratification and weighting via the propensity score in estimation of causal treatment effects: a comparative study. Stat Med. 2004; 23(19): 2937-6
