# Supplementary material for: The impact of urban regeneration programmes on health and health-related behaviour: Evaluation of the Dutch District Approach 6.5 years from the start
Source: PLoS One. 2017 May 9;12(5):e0177262. doi: 10.1371/journal.pone.0177262 (PMC5423649; doi:10.1371/journal.pone.0177262)
Supplement: S1 Table — (DOCX) [file pone.0177262.s002.docx]

**S1 Table Type of activities, level of impact, and criteria for a smaller, intermediate, or larger scale of the combined activities from the Dutch District Approach**

**Table 1 Type of activities, level of impact, and criteria for a smaller, intermediate, or larger scale of the combined activities. Source: Droomers et al., 2014**

| **Type of action** |  | **Scale** | | |
| --- | --- | --- | --- | --- |
|  | **Level^a^** | **Smaller or none** | **Intermediate** | **Larger** |
| **Employment and income** |  |  |  |  |
| Unemployment | P | <1% residents | 1%–5% residents | ≥5% residents |
| Local economic activities | N | No intervention | 1–4 businesses | ≥5 businesses |
| Debt assistance and tax reductions | P | <1% residents | 1%–5% residents | ≥5% residents |
| **Education** |  |  |  |  |
| Broad-based (primary) schools^b^ | U | <1% 4–12 years | 1%–5% 4–12 years | ≥5% 4–12 years |
| School dropout prevention | P | <1% 12–20 years | 1%–5% 12–20 years | ≥5% 12–20 years |
| **Housing** |  |  |  |  |
| Housing quality | U | <1% houses | 1%–5% houses | ≥5% houses |
| Housing stock | N | <1% houses | 1%–5% houses | ≥5% houses |
| **Physical neighbourhood environment** |  |  |  |  |
| Public parks and gardens | U | No intervention | Verges or shrubberies | Parks |
| Footpaths or bicycle paths | U | No intervention | 1–4 paths | ≥5 paths |
| Playgrounds and facilities | U | No intervention | 1–4 playgrounds | ≥5 playgrounds |
| Sports facilities and activities | U | No intervention | Facilities or activities (<5% participation) | Facilities and activities (≥5% participation) |
| **Social neighborhood environment** |  |  |  |  |
| Social cohesion, support and capital | N | 25% residents | < 25%–50% residents | ≥50% residents |
| **Social safety** |  |  |  |  |
| Nuisance and conflicts | N | No intervention | Passive (e.g., cameras) | Active (e.g., supervision) |
| Harassment by young people | N | No intervention | Youth leisure activities | Individual programs |
| Tidiness (vandalism, graffiti, trash, decay) | N | No intervention | Extra facilities | Active cleaning service |
| Burglary | N | No intervention | Information campaigns | Preventive measures |
| Traffic safety | N | No intervention | 1–4 streets | ≥5 streets |

^a^ Three levels of impact: P = interventions impacting participants, U = neighborhood changes affecting users and N = neighborhood changes affecting all residents.

^b^ For the scale of broad-based primary schools, we retrieved the number of pupils attending those schools, assuming that they all lived in the deprived district where the schools were located.
