# Supplementary material for: The impact of urban regeneration programmes on health and health-related behaviour: Evaluation of the Dutch District Approach 6.5 years from the start
Source: PLoS One. 2017 May 9;12(5):e0177262. doi: 10.1371/journal.pone.0177262 (PMC5423649; doi:10.1371/journal.pone.0177262)
Supplement: S2 Table — (DOCX) [file pone.0177262.s003.docx]

**S2 Table Comparison of health and health-related behaviour in low intensity target districts, high intensity target districts and control districts**

**Comparison of health and health-related behaviour between 2003-mid 2008 and mid 2008-2014 (general health, smoking, overweight and obesity) and between 2004-mid 2008 and mid 2008-2013 (mental health, leisure-time walking and cycling, and weekly sports) in 19 target districts intervening with high intensity and 17 districts with low intensity, and the control districts**

|  |  | **Target districts** | | | **Control districts** | |  | |  |  |  |
| --- | --- | --- | --- | --- | --- | --- | --- | --- | --- | --- | --- |
|  |  | **Pre-intervention** | **Intervention** | **Intervention versus pre-intervention^a^** | **Pre-intervention** | **Intervention** | | **Intervention versus pre-intervention^a^** | | **DiD (C.I.)^c^** | **p-value** |
| **District type** | ***n^b^*** | ***%*** | ***%*** | ***%*** | ***%*** | ***%*** | | ***%*** | |  |  |
| **Good general health** |  |  |  |  |  |  | |  | |  |  |
| High-intensity districts | 3,547 | 70.2 | 68.1 | -2.1 | 74.3 | 70.7 | | -3.6 | | 1.4 (-4.3;7.2) | 0.63 |
| Low-intensity districts | 1,708 | 66.8 | 62.5 | -4.3 | 73.6 | 72.8 | | -0.8 | | -3.4 (-11.8;4.9) | 0.42 |
| **Fair or good mental health** |  |  |  |  |  |  | |  | |  |  |
| High-intensity districts | 1,846 | 83.9 | 84.5 | 0.6 | 87.9 | 83.6 | | -4.3 | | 4.9 (-1.6;11.4) | 0.14 |
| Low-intensity districts | 922 | 81.3 | 82.2 | 0.9 | 88.7 | 88.2 | | -0.5 | | 1.4 (-7.8;10.5) | 0.77 |
| **Leisure-time walking** |  |  |  |  |  |  | |  | |  |  |
| High-intensity districts | 1,953 | 61.4 | 67.5 | 6.1 | 61.2 | 63.9 | | 2.7 | | 3.4 (-5.1;12.0) | 0.43 |
| Low-intensity districts | 965 | 58.2 | 63.8 | 5.6 | 60.0 | 62.3 | | 2.3 | | 3.3 (-9.1;15.7) | 0.60 |
| **Leisure-time cycling** |  |  |  |  |  |  | |  | |  |  |
| High-intensity districts | 1,960 | 40.4 | 47.3 | 6.9 | 48.0 | 50.3 | | 2.3 | | 4.6 (-4.2;13.4) | 0.30 |
| Low-intensity districts | 951 | 35.9 | 48.1 | 12.2 | 45.8 | 50.9 | | 5.1 | | 7.1 (-5.8;20.0) | 0.28 |
| **Sport participation** |  |  |  |  |  |  | |  | |  |  |
| High-intensity districts | 1,983 | 39.1 | 40.0 | 0.9 | 45.0 | 43.2 | | -1.8 | | 2.6 (-5.7;11.0) | 0.54 |
| Low-intensity districts | 983 | 37.9 | 36.1 | -1.8 | 45.6 | 39.5 | | -6.1 | | 4.4 (-7.7;16.4) | 0.48 |
| **Overweight** |  |  |  |  |  |  | |  | |  |  |
| High-intensity districts | 3,075 | 43.6 | 49.6 | 6.0 | 40.8 | 45.6 | | 4.8 | | 1.2 (-5.8;8.3) | 0.73 |
| Low-intensity districts | 1,548 | 40.3 | 53.5 | 13.3 | 40.2 | 48.5 | | 8.3 | | 4.9 (-4.8;14.6) | 0.32 |
| **Obesity** |  |  |  |  |  |  | |  | |  |  |
| High-intensity districts | 3,075 | 13.9 | 15.1 | 1.2 | 10.2 | 12.8 | | 2.6 | | -1.4 (-6.2;3.4) | 0.57 |
| Low-intensity districts | 1,548 | 14.8 | 17.2 | 2.4 | 12.4 | 13.1 | | 0.7 | | 1.7 (-5.3;8.6) | 0.64 |
| **Smoking** |  |  |  |  |  |  | |  | |  |  |
| High-intensity districts | 3,459 | 34.0 | 32.0 | -2.0 | 34.7 | 31.2 | | -3.5 | | 1.5 (-4.7;7.7) | 0.64 |
| Low-intensity districts | 1,234 | 34.3 | 30.8 | -3.5 | 35.2 | 32.2 | | -3.0 | | -0.5 (-9.4;8.4) | 0.91 |

^a^ Reference category; ^b^ The n is the sum of all four groups used in the analysis; ^c^ Difference in Difference (Confidence Intervals)
