# Supplementary material for: The impact of urban regeneration programmes on health and health-related behaviour: Evaluation of the Dutch District Approach 6.5 years from the start
Source: PLoS One. 2017 May 9;12(5):e0177262. doi: 10.1371/journal.pone.0177262 (PMC5423649; doi:10.1371/journal.pone.0177262)
Supplement: S3 Table — (DOCX) [file pone.0177262.s004.docx]

**S3 Table Comparison of health and health-related behaviour between the pre-intervention period and the early and late intervention period**

**Comparison of health and health-related behaviour between 2003-mid 2008, mid 2008-2011 and 2012-2014 in the 40 target districts and the control districts**

|  |  | **Target districts** | | | **Control districts** | |  |  | |  | |
| --- | --- | --- | --- | --- | --- | --- | --- | --- | --- | --- | --- |
|  |  | **Pre-intervention** | **Intervention early period** | **Intervention late period** | **Pre-intervention** | **Intervention early period** | **Intervention late period** | **Intervention early period versus pre-intervention^a^** | **p-value** | **Intervention late period versus pre-intervention^a^** | **p-value** |
|  | | ***2003-mid 2008*** | ***mid 2008 -2011*** | ***2012-2014*** | ***2003-mid 2008*** | ***mid 2008 -2011*** | ***2012-2014*** |  |  |  |  |
| ***outcome*** | ***n^b^*** | ***%*** | ***%*** | ***%*** | ***%*** | ***%*** | ***%*** | **DiD (C.I.)^c^** |  | **DiD (C.I.)^c^** |  |
| Good general health | 9,900 | 68.0 | 64.1 | 68.6 | 74.3 | 73.3 | 72.8 | -2.9 (-9.2;3.4) | 0.37 | 2.1 (-4.7;8.8) | 0.55 |
| Fair or good mental health | 5,162 | 82.8 | 82.0 | 85.3 | 86.6 | 85.6 | 84.4 | -0.3 (-7.2;7.7) | 0.95 | 4.7 (-3.2;12.6) | 0.24 |
| Leisure-time walking | 5,679 | 60.1 | 65.0 | 62.9 | 60.4 | 65.8 | 66.4 | -0.4 (-9.7;8.8) | 0.92 | -3.2 (-13.4;6.9) | 0.53 |
| Leisure-time cycling | 4,915 | 41.1 | 42.7 | 52.8 | 47.3 | 51.5 | 55.7 | -2.6 (-12.7;7.5) | 0.61 | 3.4 (-8.4;15.2) | 0.57 |
| Sport participation | 5,476 | 40.3 | 36.9 | 38.7 | 45.6 | 41.3 | 43.8 | 0.9 (-8.4;10.2) | 0.85 | 2.0 (-8.3;12.3) | 0.70 |
| Overweight | 8,499 | 45.2 | 49.8 | 50.3 | 41.2 | 45.3 | 43.8 | 0.5 (-6.9;7.8) | 0.90 | 2.4 (-5.6;10.4) | 0.56 |
| Obesity | 8,499 | 14.9 | 13.5 | 15.8 | 10.7 | 12.6 | 12.1 | -3.3 (-8.2;1.7) | 0.19 | -0.5 (-6.0;5.0) | 0.85 |
| Smoking | 9,140 | 33.7 | 34.4 | 29.2 | 35.1 | 34.6 | 30.4 | 1.2 (-5.8;8.2) | 0.74 | 0.2 (-7.4;7.7) | 0.95 |

^a^ Reference category; ^b^ The n is the sum of all four groups used in the analysis; ^c^ Difference in Difference (Confidence Intervals)
